# Ordinal-To-Interval Scale Conversion Tables and National Items for the New Zealand Version of the WHOQOL-BREF

**DOI:** 10.1371/journal.pone.0166065

**Published:** 2016-11-03

**Authors:** Christian U. Krägeloh, D. Rex Billington, Patricia Hsien-Chuan Hsu, Xuan Joanna Feng, Oleg N. Medvedev, Paula Kersten, Jason Landon, Richard J. Siegert

**Affiliations:** 1 Auckland University of Technology, Auckland, New Zealand; 2 University of Brighton, Brighton, United Kingdom; TNO, NETHERLANDS

## Abstract

The World Health Organisation Quality of Life (WHOQOL) questionnaires are widely used around the world and can claim strong cross-cultural validity due to their development in collaboration with international field centres. To enhance conceptual equivalence of quality of life across cultures, optional national items are often developed for use alongside the core instrument. The present study outlines the development of national items for the New Zealand WHOQOL-BREF. Focus groups with members of the community as well as health experts discussed what constitutes quality of life in their opinion. Based on themes extracted of aspects not contained in the existing WHOQOL instrument, 46 candidate items were generated and subsequently rated for their importance by a random sample of 585 individuals from the general population. Applying importance criteria reduced these items to 24, which were then sent to another large random sample (*n* = 808) to be rated alongside the existing WHOQOL-BREF. A final set of five items met the criteria for national items. Confirmatory factor analysis identified four national items as belonging to the psychological domain of quality of life, and one item to the social domain. Rasch analysis validated these results and generated ordinal-to-interval conversion algorithms to allow use of parametric statistics for domain scores with and without national items.

## Introduction

The World Health Organisation defined health as “a state of complete physical, mental and social well-being and not merely the absence of disease and infirmity” (p.1315) [1]. Unlike traditional indicators of health, such as mortality, morbidity, and clinical appraisals [2], the WHO’s definition of health implies that assessment of health and effects of health care should not only include an indication of changes in frequency and severity of symptoms but also an estimation of quality of life (QOL) [3]. In the last two to three decades, the increasingly wide-spread use of health-related QOL (HRQOL) instruments reflects this appreciation of the need to gather the patient’s perspective of their wellbeing.

The WHO defined QOL as “individuals’ perception of their position in life in the context of the culture and value systems in which they live, and in relation to their goals, expectations, standards, and concerns” (p.1405) [4]. In 1991, the WHO initiated an international collaborative project to develop a generic cross-culturally valid measure of HRQOL [5]. Representing diverse cultures around the world, 15 collaborative centres in 14 countries contributed data to this project. This included qualitative data from focus groups that inquired which facets of QOL were considered important by individuals, as well as quantitative data from testing the suitability of candidate items that had been developed from the emerging focus group themes. The resulting WHOQOL-100 instrument contains 24 facets that yield a six-domain profile of QOL: physical QOL, psychological QOL, level of independence, social relationships, environmental QOL, and spiritual/religious and personal beliefs [3]. Each of the 24 facets is assessed using four questions, and an additional general facet contains another four questions on overall QOL or health. Tests of the universality of the WHOQOL-100 support the assertion that the WHOQOL is applicable and comparable across cultures [6]. To reduce response burden, the WHOQOL-100 was later shortened to a 26-item version named the WHOQOL-BREF [7]. The WHOQOL-BREF contains one item from each of the 24 WHOQOL-100 facets and two global items each of QOL and health.

Even though the WHOQOL was developed by collaborating centres from a range of countries, there could be instances where items do not fully cover the entire range of important aspects of HRQOL for a particular culture, in which case additional national items are permitted [3]. By including national items, relevant facets for certain cultural groups could be included, thus improving the conceptual equivalence of QOL. Of the 15 original WHOOQL field centres, nine centres (Bangkok, Bath, Beer Sheva, Harare, Madras, New Delhi, St. Petersburg, Tokyo, and Zagreb) selected national items [8]. The subsequently established WHOQOL centres in Mainland China, Hong Kong, and Taiwan also selected national items [8–10]. One of the 12 national items of the Hong Kong version, for example, was “How satisfied are you with your destiny?” and another one “How is your appetite?” [9].

The WHOQOL-BREF has previously been validated for use in New Zealand samples from the general population [11], but more precision may be gained if facets relevant to New Zealand are also included. The purpose of the present study was to investigate to what extent the WHOQOL captures the range of facets considered important by New Zealanders for their QOL and to develop national items if necessary. This investigation proceeded in three stages. Study 1 involved 12 focus groups that explored the range of QOL issues deemed important by New Zealanders. This produced 16 themes beyond those already contained in the generic WHOQOL, which then led to the development of 46 potential national items. Study 2 was a postal survey with a large sample from the general population that asked respondents to rate the importance of the existing WHOQOL facets as well as the facets expressed in the 46 candidate national items. Of these, a set of 24 items met several importance criteria and was selected for further testing. In Study 3, these 24 items and the WHOQOL-BREF were sent as a postal survey to another large sample of individuals randomly selected from the general population. Only 5 of the 24 items met the WHOQOL criteria for suitable national items. A series of analyses including ordinal-level confirmatory factor analysis then tested the psychometric properties of the instrument with these new national items. Finally, Rasch analysis confirmed these results and provided ordinal-to-interval conversion algorithms for the domain scores with and without national items. An ordinal scale will not become an interval scale simply because of its popularity or by adding individual Likert-scale items scores together [12,13]. Using the ordinal-to-interval conversion tables presented here will increase precision of WHOQOL domains and permit statistical analysis without the need to break assumptions of parametric statistics.

## Study 1 (Focus Groups)

The purpose of this study was to investigate which aspects New Zealanders consider important to their QOL and particularly any aspects that are currently not assessed by the WHOQOL. Such additional facets would then be developed into importance questions to be tested for their potential to be suitable New Zealand national WHOQOL items.

### Method for Study 1

#### Participants and Procedure

Recruitment for focus groups occurred between November 2008 and May 2009. Participants were recruited using a variety of different methods. Advertisements were distributed in university newsletters and noticeboards as well as by directly approaching various community groups and local and national organisations providing health services. Participants were also recruited through word-of-mouth, snowballing, e-mail lists, universities, and health rehabilitation institutes. In total, 12 focus groups were conducted. Each focus group was intended to have six to eight individuals. However, due to late cancellations, some of the focus groups contained smaller numbers, the lowest being three. The total number of participants was 61, and efforts were made to ensure that the participants were demographically representative of New Zealand population in terms of gender, age, marital status, educational background, and ethnic identity. Four groups were outpatients with chronic health conditions and informal caregivers, three groups consisted of health professionals and academics working in health fields, one group comprised of members from the general population with no specifically identified health condition, one group consisted of university students, two groups were older adults, and one group consisted of recent immigrants to New Zealand.

The focus groups were conducted by two of the researchers following the standard protocol recommended by the WHOQOL Group [14]. Each focus group started with a brief introduction to the purpose of the research as well as handing out and explanation of relevant documents such as participant information sheets and consent forms. Participants were then asked to describe and discuss what they thought was important to their QOL and that of New Zealanders. Following that, discussions took place about what participants thought were important determinants of the QOL of people who were not well. Lastly, participants were given the existing WHOQOL facets to rate for their importance to QOL. The focus groups sessions lasted between 90 and 120 minutes and were audio taped. One of the researchers also took notes during the focus groups. Participants were provided with a detailed information sheet about the study and provided written consent to participate. The study was approved by the Auckland University of Technology Ethics Committee.

#### Data Analysis

After all the focus groups were completed, the researchers met on repeated occasions in groups of three to listen to the tapes, take notes, discuss the content of the tape recordings, and compare notes. The purpose of these meetings was to discern possible facets of QOL that could be tested for their generality later on. Sometimes the tape was stopped to permit clarification and discussion of points being made by participants. Any QOL facets that arose from the focus group discussions that were not in the existing WHOQOL were identified as new themes in this study. Consensus among the research team was not necessary: A potential facet could be proposed by an analyst even if the other analysts did not agree. However, this did not occur. Most of the new themes that were proposed in this study were revealed in at least three different focus groups. The research team then proposed and composed importance questions, based on the identified new themes. The resulting list of new items was then presented to two English language experts to review for grammar and comprehensibility, which resulted in some small changes.

### Results and Discussion

Detailed results for the various focus groups are presented elsewhere [15]. Overall, all groups participated in an enthusiastic manner and contributed to interesting and rewarding discussions. All participants accepted the WHO’s definition of QOL and saw its relevance to human experience. Much of the discussions centered around different nuances expressed in the existing WHOQOL facet, although almost all of the existing WHOQOL facets were rated as important to a person’s QOL. Many of the new themes that emerged were raised by the different types of focus groups (outpatients, health experts, general population, older adults, and recent immigrants). The recent immigrant focus group was slightly different in the sense that a large amount of discussion was dedicated to comparing their country of origin with New Zealand. Such discussion was generally about medical care and education systems, as prompted by some of the existing WHOQOL facets. Aspects emphasised in this focus group were *acceptance of one’s culture* and *having freedom to do things*.

Table 1 presents the 16 themes generated from all focus group discussions of aspects that are currently not covered in the WHOQOL. Some of these themes overlap with themes of facets of the WHOQOL but capture additional aspects. For example, the theme *Home environment* contained aspects of privacy related to one’s home environment. Other aspects were completely new, such as *Nature and outdoors*. This theme has emerged quite strongly and relates to having access to nature and ability to engage in outdoor activities, such as by visiting a beach or hiking in the bush.

**Table 1:** Summary of the themes generated from the focus group discussions of aspects currently not covered in the WHOQOL. Themes are listed in alphabetical order.

Family and children
Freedom of expression, autonomy, choice
Food and diet
Health and social care
History, roots and memories
Home environment
Identity and belonging
Managing personal difficulties and disabilities
Modern technology
Nature and outdoors
Opportunities to acquire new information
Physical fitness
Purpose and goals
Stability
Stigma, respect, prejudice, multiculturalism, diversity
Trust, security and crime

Based on the 16 new themes listed in Table 1, importance items were developed in preparation for the next phase of the project that would confirm the importance of the new facets in a large quantitative study (Study 2). Items were written in the format “How important to you is …?”. For most of the themes, more than one item was generated to allow testing of various alternatives that may have slightly different nuances and connotations. In total, 46 such new items were developed. Many of these items appeared to belong to existing WHOQOL domains based on face validity. However, alignment with domains was not a criterion for item development, and items were written that could potentially sit outside the existing domain structure.

Some limitations of the focus groups need to be acknowledged. Firstly, due to illness and other unforeseen circumstances, two of the focus groups only had three participants, thus somewhat limiting the richness of discussions. Secondly, the research was conducted during a time when a recent legislative change was being debated in New Zealand. Dubbed by community groups and the media as the *anti-smacking law*, a legislative change in 2007 repealed a section from the Crimes Act 1961, which provided a statutory defence for adults prosecuted for assaulting a child if it was parental force for the use of correction [16]. Various lobby groups were strongly opposed to the *anti-smacking law* and forced a citizen-initiated referendum in 2009, which happened to occur around the time this research was being conducted. Participants in the present study made occasional references to government control and related issues. Some of this discussion may thus have been a reflection of the political circumstances at the time rather than a representation of relatively stable and long-term perceptions of QOL.

## Study 2 (Importance of WHOQOL Facets)

The purpose of this study was to assess to what extent the existing WHOQOL facets including those new emerging themes from Study 1 (Table 1) are judged by New Zealanders as important to their QOL. Using a large sample of the general population, importance ratings of the 46 items generated in Study 1 were rated alongside the existing WHOQOL items.

Importance ratings of the existing WHOQOL items were published elsewhere [17] and will not be presented here. Krägeloh et al. [17] compared WHOQOL importance ratings of New Zealanders with ratings from participants in Australia and other countries around the world. The present study focuses on importance ratings of the 46 items generated in Study 1 and how a final set of candidate items was selected for further testing.

### Method

#### Participants and Procedure

Detailed information about recruitment and the participants is presented elsewhere [17]. Using a random sample of the general population, 2,000 questionnaires were posted by mail with a pre-paid return envelope. Participants rated the importance of 46 items developed in Study 1 in the format “How important to you is …?”. Response options were on a 5-point Likert scale ranging from 1 = “not important” to 1 = “extremely important”. These items were presented together with a section requesting demographic information and a section that asked participants to rate the importance of the 26 existing items of the WHOQOL-BREF. Two versions of the questionnaire with different question ordering were sent out to control for order effects. The number of valid returned questionnaires was 585, thus a response rate of 29%.

#### Data analysis

Analyses were conducted using the software Statistical Package for the Social Sciences (SPSS). The selection criteria for candidate national items were that the mean importance rating had to be 4 or greater on the 5-point scale or a mean marginally below 4 but with a median value of at least 4. Inferential statistics were used to examine the relationship between importance ratings and the demographic variables gender, age, and self-reported health state. The percentage of missing data was 0.001.

### Results and Discussion

The overall mean of the importance ratings for existing WHOQOL questions was 4.07. For most items, mean importance ratings were above 4 or close to 4. Exceptions were *Chances of getting new information and knowledge* (3.61), *Bodily image and appearance* (3.54), *Support from others* (3.50), and *Sexual life* (3.09). For the 46 candidate items, the mean importance ratings ranged from 3.07 (*Having diverse culture*) to 4.61 (*Immediate family*). Other items with very low scores were *Living in a multicultural society* (3.18) and *Being part of a group* (3.22).

Ten of the 46 items were rated significantly differently by individuals who considered themselves as ill versus those considering themselves well. For 18 items, there were significant differences in rating by age, and for 36 items there were gender differences. WHOQOL importance ratings are known to be different by these demographic variables [17], and in this case no particular pattern was evident. The main criterion applied to reduce the number of items was that mean ratings were at least 4, or when close to 4 that the median was 4. Additionally, when considering items with similar meanings, such as *Good healthy food*, *Eating sensibly*, and *Proper diet*, the item with the highest importance rating was chosen and the other discarded. Another example includes *Have natural surroundings*, *New Zealand natural environment*, and *Access to outdoor activities*. This way, the number of items was reduced from 46 to 24.

In preparation for Study 3 (testing the psychometric properties of national items), importance items were now re-written in the standard WHOQOL item format of either intensity (e.g., “How much do you…?”), frequency (e.g., “How often do you have…?”), capacity (e.g., “How well are you able to…?”), or evaluation (e.g., “To what extent do you feel…?”). This writing was done by one of the researchers and edited by two independent advisors, one being a lawyer and another one a journalist. The research team then examined the written questions and chose the best format for each item. Some items that closely related to a particular cultural issue in New Zealand were altered slightly in order to accentuate cultural aspects. For example, the item asking about the importance of one’s immediate family was reformatted into “How satisfied are you with the support you get from your family/whānau?” to capture the special meaning of the Māori word *whānau* (extended family). Another item was edited to achieve greater generalisability to all people throughout their lifespan and not just a particular time in their life. For example, the importance item asking “How important to you is having high quality education available?” could be interpreted as only applying to an individual’s personal education needs. This item was rephrased to “How satisfied are you with the quality of education that is available?”, which means that it could apply to others the individual is involved with. Table 2 lists the final set of 24 items in their original importance format and the reworded WHOQOL question format to be tested in Study 3.

**Table 2:** The final list of 24 facets to be tested further. On the left-hand side are the QOL facets worded as importance items, which were used in Study 2. On the right-hand side are newly worded WHOQOL questions to be tested for their psychometric properties in Study 3.

Original importance item	Item in WHOQOL question format
How important to you is individual freedom?	N1: To what extent do you feel you have individual freedom?
How important to you is being successful in what you choose to do?	N2: To what extent do you feel successful in the things you choose to do?
How important to you is feeling you have control over your life?	N3: To what extent do you feel you have control over your life?
How important to you are your family roots?	N4: How much do you value your family roots?
How important to you is it that others have a sense of responsibility?	N5: To what extent do you feel others have a sense of responsibility?
How important to you is being trusted by others?	N6: To what extent do you feel trusted by others?
How important to you is your privacy?	N7: To what extent do you feel you have enough privacy?
How important to you is having access to competent medical personnel?	N8: To what extent do you feel you have access to competent medical personnel?
How important to you is a feeling of belonging?	N9: To what extent do you have feelings of belonging?
How important to you is respect for your culture?	N10: To what extent do you feel your culture is respected?
How important to you is being able to manage personal difficulties?	N11: To what extent are you able to manage personal difficulties?
How important to you is a fair and just society?	N12: To what extent do you feel you live in a fair and just society?
How important to you is being able to trust others?	N13: To what extent do you feel you can trust others?
How important to you is New Zealand’s natural environment?	N14: To what extent do you enjoy New Zealand’s natural environment?
How important to you is being accepted for who you are?	N15: To what extent do you feel accepted for who you are?
How important to you is being respected by others?	N16: To what extent do you feel respected by others?
How important is it to you to have government listen to citizens’ views?	N17: How satisfied are you that the government listens to citizens’ views?
How important to you is your immediate family?	N18: How satisfied are you with the support you get from your family/whānau?
How important to you is good healthy food?	N19: How satisfied are you that you eat healthily?
How important to you is having high quality education available?	N20: How satisfied are you with the quality of education that is available?
How important to you is being able to meet the expectations of others?	N21: How satisfied are you that you are able to meet the expectations placed on you?
How important to you is physical exercise?	N22: How satisfied are you that you get enough physical exercise?
How important to you is living in a society that accepts differences among people?	N23: How satisfied are you that you live in a society that accepts differences among people?
How important to you is having access to modern technology?	N24: How satisfied are you with your access to modern technology?

Overall, the results from this quantitative study confirm the relevance of the themes brought up in the focus groups of Study 1. The range of the mean importance ratings of the 46 importance items (3.07 to 4.61) that were derived from the focus group themes was comparable with that of the existing WHOQOL items, which was 3.09 to 4.50 [17]. Nevertheless, stringent criteria were applied for the selection of candidate national items even if that meant discarding items with importance ratings that were higher than some of the ratings of the existing WHOQOL items. Given the previous work demonstrating the universality of the WHOQOL facets and the cross-cultural validity of the scale [6], the present study aimed to leave the core instrument in its original format. Instead, however, the precision of measuring QOL in New Zealand may be enhanced by careful selection of national items and thus inclusion of facets that are considered particularly important to New Zealanders. To achieve such increased precision, it was necessary to include only items that were clearly considered suitable. The subsequent psychometric testing in Study 3 aimed to reduce the number of items even further as 24 optional national items are not practical. The abbreviated 26-item WHOQOL-BREF is attractive because of its reduced response burden compared to the WHOQOL-100 –an advantage that would be eliminated if almost the same number of national items were added.

## Study 3 (Psychometric Testing of New National Items and Ordinal-to-Interval Conversion Tables)

The purpose of this study was to test the psychometric properties of the 24 candidate national items developed in Study 2 in order to determine a final set of national items. Analyses also investigated to which domains these new items need to be assigned, and lastly, ordinal-to-interval conversion tables were produced using Rasch analysis to enable use of domain scores in parametric statistics.

### Method

#### Participants and Procedure

Detailed information about participants and procedure is published elsewhere [11]. Using the national electoral roll, 3,000 individuals from the general population were randomly selected and sent a mail survey with a pre-paid return envelope. The questionnaire contained the 26 items of the core WHOQOL-BREF instrument as well as the 24 items developed in Study 2 (Table 2) and a demographics section. Two versions of the questionnaire with different question ordering were used to control for order effects. Due to underrepresentation of younger persons, a supplementary sample of younger adults was obtained using purposive sampling [11]. The total number of valid questionnaires was 808.

#### Data analysis

Inferential statistical analyses were conducted using SPSS. Based on previous WHOQOL studies [8,9], the following criteria were used to determine the suitability of national items:

- Item means are between 2 to 4 on the 5-point Likert scale.
- Correlations of the item are above 0.30 with a) total QOL, b) the *g* facet (sum of the global QOL and health items), and c) the global QOL and health items separately.
- The item discriminates between ill and well participants.
- The item does not duplicate any of the other candidate national items.
- The item does not duplicate any of the existing WHOQOL items.
- Correlation above 0.40 with the likely parent domain (physical, psychological, social or environmental QOL) of the item.
- The overall Cronbach’s alpha is above 0.70 at the domain level with candidate national item added, and overall Cronbach’s alpha decreases when candidate national item is removed.
- The national items significantly predict total QOL when other existing WHOQOL items from the same domain are controlled for.
- A factor structure with the national items provides an acceptable fit.
- An item has an acceptable fit to the Rasch model (fit residuals ±2.5).

Factor structure was assessed using confirmatory factor analysis. Here, asymptotic distribution-free confirmatory factor analysis was conducted using LISREL v. 8.80 [18] in the same way as outlined by Krägeloh et al. [11]. Three indices were used to evaluate goodness of fit: root mean square error of approximation (RMSEA), comparative fit index (CFI) and standardised root mean square residual (SRMR). Following frequently quoted guidelines [19], model fits were considered acceptable if RMSEA < 0.06, CFI > 0.90, and SRMR < 0.08. Subsequent Rasch analysis using the software RUMM2030 [20] was conducted to confirm the results by exploring item functioning and the unidimensionality of each domain. The approach taken in the Rasch analysis is outlined in great detail elsewhere [21]. Provided the data fits the Rasch model, the present study aimed to produce ordinal-to-interval conversion algorithms to allow the use of domain scores (original domain scores and domain scores with optional national items) without the need to break fundamental assumptions of parametric statistics.

### Results and Discussion

The means of the 24 candidate national items (N1 to N24, see Table 2) ranged from 2.65 (*Government listens to views*) to 4.20 (*Value family roots*), and most were between 3.00 and 4.00. Most items had acceptable levels of skewness kurtosis, with only two items (*Value family roots* and *Access modern technology*) marginally outside the normal range of -1.00 to 1.00 [22] for skewness, and two items (*Access modern technology* and *Meet expectations*) marginally outside the range for kurtosis.

The following analyses investigated concurrent validity by correlating each national item with a total QOL score and the score on the *g* facet. Item N4 had an item-total and item-*g* facet correlation of less than 0.30. This item was therefore discarded, together with items N5, N6, N7, N14, N17, N18, N20 and N23, which had correlations of less than 0.30 with the *g* facet. The remaining items N1, N2, N3, N8 N9, N10, N11, N12, N13, N15, N16, N19, N21, N22, and N24 were then examined for their correlations with the two general items (global QOL and global health) of the WHOQOL-BREF. Only items N1, N2, N3, N9, N11, N16, N19 and N21 had correlations over 0.30 with both general items and were thus retained.

The next step was to examine the discriminant validity of the candidate national items that had met the above criteria for concurrent validity. When controlling for gender, the scores for ill people were significantly (*p* < .01) different from those of well people, except for item N19 (*Eat healthily*) (*F*(1, 788) = 3.52, *p>*.05), which was then discarded. Correlation coefficients were calculated for the remaining seven candidate national items (N1, N2, N3, N9, N11, N16, and N21) to investigate whether there are any item duplications. Items N1 (*Individual freedom*), N2 (*Successful in things*), and N3 (*Control over life*) were identified as having coefficients above 0.60. Since N3 had the highest correlations of all three items with the global QOL and health items as well as the *g* facet, N3 was retained and the redundant items N1 and N2 were discarded. Finally, correlation coefficients were calculated for the remaining five items (N3, N9, N11, N16, and N21) to assess whether these candidate items duplicate any of the existing WHOQOL items. As none of the correlations were above 0.60, this was not the case. A final semantic check of the items also verified that none of the remaining five candidate national items covered the same content as the existing WHOQOL items.

Items N3, N9, N11, N16, and N21 also met the criterion of correlations above 0.40 with domain scores. All items were most highly correlated with the psychological domain (range 0.58 to 0.63). While Item N9 had a marginally higher correlation (0.60) with the psychological domain than with the social domain (0.55), this item (*Feelings of belonging*) appeared to belong (no pun intended) more to the social domain based on face validity. However, the subsequent confirmatory factor analysis did explore the assignment of this item to either of the two domains.

Cronbach’s alpha for the items of the psychological domain of the WHOQOL-BREF and the four proposed national items for this domain (N3, N11, N16, and N21) was 0.89, which indicated good internal consistency. The corrected item-total correlation for the four proposed national items ranged from 0.61 to 0.65, suggesting that the proposed national items were consistent with the WHOQOL-BREF psychological items. Each potential national item made a positive contribution to the reliability of the psychological domain, as demonstrated by the decreased overall Cronbach’s alpha when each national item was deleted. Similarly, Item N9 met the reliability criteria for the social domain. Cronbach’s alpha with Item N9 was 0.76, indicating adequate internal consistency. The corrected item-total correlation was 0.55 and thus above the cut-off value of 0.40, and Cronbach’s alpha decreased to 0.72 if the item is removed.

A series of hierarchical multiple linear regressions were conducted to examine the prediction validity of the candidate national items. In the case of the psychological domain, Items N3, N11, N16, and N21 significantly predicted the total QOL score when existing items of the psychological domain were controlled for in the first block of the regression. Standardised beta coefficients ranged from 0.05 to 0.12. Similarly, Item N9 significantly predicted (standardised beta coefficient of 0.32) total QOL when the other social domain items were controlled for.

After the above-mentioned criteria have been applied, a final set of five national items remained: N3 (“To what extent do you feel you have control over life?”), N9 (“To what extent do you have feelings of belonging?”), N11 (“To what extent are your able to manage personal difficulties?”), N16 (“To what extent do you feel respected by others?”), and N21 (“How satisfied are you that you are able to meet the expectations placed on you?”). Table 3 provides a summary of correlations of the national items with the criterion variables, as discussed above.

**Table 3:** National item means and correlations (Pearson’s r) with criterion variables, namely total QOL score, g facet (sum of the global QOL and health items), global QOL item, global health item, and parent domain total). The parent domain was the psychological domain for items N3, N11, N16, and N21 and the social domain for N9. The item to parent domain coefficients were calculated with domain scores when the national items had not been added.

Final national items	Mean	Item to total QOL	Item to *g* facet	Item to global QOL item	Item to global health item	Item to parent domain
N3. Control over life	3.79	.63**	.47**	.47**	.36**	.63**
N9. Feelings of belonging	3.77	.61**	.40**	.39**	.32**	.55**
N11. Manage personal difficulties	3.78	.62**	.45**	.44**	.36**	.60**
N16. Respected by others	3.72	.59**	.38**	.35**	.31**	.63**
N21. Meet expectations	3.78	.59**	.46**	.40**	.41**	.58 **

** *p* < .01

The adequacy of the proposed factor solutions was tested using confirmatory factor analysis. A diagonally-weighted least-squares confirmatory factor analysis with polychoric correlations tested three alternative models. Model A was the original WHOQOL-BREF domain structure without the national items. Model B assigned all five national items to the psychological domain, which was the domain that these items had the highest correlations with. Model C assigned all national items to the psychological domain except for the national item N9 (*Feelings of belonging*), which was assigned to the social relationships domain based on face validity. Both Models B (RMSEA = 0.067, CFI = 0.974, SRMR = 0.067) and C (RMSEA = 0.066, CFI = 0.974, SRMR = 0.065) provided improved fit indices over the original WHOQOL-BREF factor structure tested in Model A (RMSEA = 0.079, CFI = 0.962, SRMR = 0.077). Model C provided the best factor structure and thus confirmed that the national item N9 is best to be assigned to the social relationships domain.

The results from Rasch analyses of the four domains of the NZ WHOQOL-BREF were reported elsewhere [11]. The present study conducted additional analyses of the fit of the psychological and social domains to the Rasch model when the new national items were added. For the psychological domain, none of the items had disordered threshold. The initial model did not fit the Rasch model, with *χ*^2^(60) = 98.04, *p* < .001. Similar to Krägeloh et al. [11], local dependency was evident, which was dealt with by creating two sub-tests. The solution resulted in an adequate fit, with *χ*^2^(42) = 41.99, *p*>.05. The adequacy of the fit was further indicated by a PSI of 0.87 (criterion >0.70) and means and standard deviations of fit residuals close to 0.00 and 1.00, respectively. Lastly, no DIF was evident, and unidimensionality was confirmed by the fact that the lower bound of a binominal confidence interval computed for the number of observed significant t tests overlapped the 5% cutoff point.

Rasch analysis of the social domain with the additional national item N9 revealed the same issues with disordered thresholds of Items 20 and 21 as reported by Krägeloh et al. [11]. Re-scoring the two items by collapsing response categories 2 and 3 resulted in an adequate fit (*χ*^2^(24) = 36.23, *p*>.05), but significant DIF was evident by age for Item 21 (*Sexual life*). Splitting the item by age improved the fit slightly, with *χ*^2^(29) = 40.63, *p*>.05 and PSI = 70. The means and standard deviations of the fit residuals were adequate, and unidimensionality of the domain was also confirmed.

Lastly, ordinal-to-interval rescoring algorithms were generated for the four WHOQOL-BREF domains without the national items as well as the psychological and social domain with the optional national items added (Table 4). Due to the DIF by age for one of the items in the social domain, separate scoring algorithms need to be applied for individuals between 18 to 60 years of age and those above 60 years. Using the conversion table presented in Table 4, the ordinal scores from adding item scores to domain sum scores can now be converted to interval-level scores, thus making the domain scores suitable for parametric statistics.

**Table 4:** Converting from ordinal- to interval-level scores for the four subscales of the New Zealand version of the WHOQOL-BREF (including and excluding optional New Zealand national items).

Physical QoL		Psychological QoL				Psychological QoL		Social QoL with national items			Social QoL without national items			Environmental QoL	
		including national items				without national items			Age 18–60	≥ 61		Age 18–60	≥ 61		
Ordinal	Interval	Ordinal	Interval	Ordinal	Interval	Ordinal	Interval	Ordinal	Interval	Interval	Ordinal	Interval	Interval	Ordinal	Interval
7	7.00	10	10.00	31	34.34	6	6.00	4	4.00	4.00	3	3.00	3.00	8	8.00
8	9.30	11	18.10	32	34.82	7	8.81	5	5.61	5.54	4	4.31	4.18	9	10.48
9	11.02	12	21.93	33	35.32	8	10.79	6	6.79	6.67	5	5.35	5.13	10	12.41
10	12.30	13	23.73	34	35.82	9	12.19	7	7.66	7.51	6	6.15	5.89	11	13.91
11	13.30	14	25.02	35	36.35	10	13.20	8	8.39	8.22	7	6.92	6.63	12	15.19
12	14.10	15	26.02	36	36.90	11	14.00	9	9.07	8.89	8	7.72	7.45	13	16.33
13	14.77	16	26.85	37	37.48	12	14.69	10	9.73	9.57	9	8.60	8.42	14	17.34
14	15.38	17	27.56	38	38.09	13	15.32	11	10.43	10.30	10	9.57	9.44	15	18.27
15	15.97	18	28.18	39	38.72	14	15.93	12	11.18	11.12	11	10.59	10.43	16	19.13
16	16.56	19	28.75	40	39.39	15	16.52	13	12.01	12.04	12	11.72	11.61	17	19.92
17	17.14	20	29.28	41	40.08	16	17.09	14	12.93	13.03	13	13.00	13.00	18	20.66
18	17.72	21	29.78	42	40.80	17	17.67	15	13.93	14.04				19	21.36
19	18.29	22	30.26	43	41.54	18	18.26	16	15.02	15.11				20	22.02
20	18.84	23	30.73	44	42.31	19	18.86	17	16.36	16.41				21	22.66
21	19.40	24	31.19	45	43.12	20	19.48	18	18.00	18.00				22	23.28
22	19.96	25	31.64	46	43.99	21	20.13							23	23.88
23	20.54	26	32.08	47	44.98	22	20.82							24	24.48
24	21.16	27	32.53	48	46.16	23	21.55							25	25.08
25	21.84	28	32.97	49	47.78	24	22.35							26	25.68
26	22.59	29	33.42	50	50.00	25	23.21							27	26.29
27	23.44	30	33.88			26	24.14							28	26.94
28	24.41					27	25.17							29	27.62
29	25.52					28	26.38							30	28.37
30	26.92					29	27.95							31	29.24
31	28.97					30	30.00							32	30.32
32	32.00													33	31.85
														34	34.00

Note: The negatively worded items W3 W4 and W26 have to be reversed coded; items W9, W12, W13, W18, W20, W21, W23, W24 and W25 should be rescored as follows: (1->1) (2->2) (3->2) (4->3) (5->4); and items W16, W17 as follows (1->1) (2->2) (3->3) (4->3) (5->4) prior to calculating domain scores. For the Physical QOL domain, add items W3, W4, W10, W15, W16, W17 and W18; for Psychological QOL with New Zealand national items, add items W5, W6, W7, W11, W19, W26, N3, N11, N16, and N21; for the Social QOL domain with New Zealand national items, add items W20, W21, W22, and N9; and for the Environmental QOL domain, add items W8, W9, W12, W13, W14, W23, W24, and W25. For each resulting subscale ordinal sum score, find the equivalent interval-level score in the above conversion table. If calculating Psychological and Social QOL scores without New Zealand national items do not include the national items in the above-mentioned summary score calculations and refer to the relevant columns above to extract the converted scores. This table cannot be used for respondents with missing data.

## General Discussion

The present paper outlined a project to develop optional national items for the New Zealand version of the WHOQOL-BREF. Using focus groups, 16 themes were identified of aspects that are not covered by existing WHOQOL facets. From the themes, 46 items were generated which were rated by a large sample of individuals from the New Zealand general population for the importance to their QOL. The number of items was then reduced to 24, which were then sent out to another large general population to be rated alongside the existing WHOQOL-BREF questions. Applying a series of criteria to test psychometric robustness, the number of items was finally reduced to five: *Control over life*, *Feelings of belonging*, *Manage personal difficulties*, *Respected by others*, and *Meet expectations*. Confirmatory factor analyses provided evidence that *Feelings of belonging* is part of the social domain, while the remaining items are best placed in the psychological domain. Rasch analysis confirmed these results and further provided ordinal-to-interval conversion algorithms that increase precision of measurement and enable the use of domain scores in parametric statistics.

Five national items were selected out of a set of 24 proposed items for the New Zealand WHOQOL-BREF questionnaire. Previously, 41 national items were selected from 138 proposed items by the 10 early established WHOQOL centres, ranging from one national item selected by the Bath, Harare and Zagreb WHOQOL centres to 10 items selected by the St Petersburg WHOQOL centre [8]. No national items were selected out of 11 items proposed by the Beer Sheva centre. The national items selected by other early established centres were 4 items (Bangkok, Tokyo), 5 items (New Delhi), 6 items (Madras) and 9 items (Hong Kong). The average number of items selected was four, which is one item less than that for the New Zealand national items. The new WHOQOL Taiwan centre has selected 12 items from 20 national items proposed for the WHOQOL-100 [9], which together with the 12 items for the Hong Kong version [10] is by far the most national items selected.

By including items unique to New Zealand culture, the conceptual equivalence of WHOQOL domains is expected to be improved for QOL comparisons across cultures. The additional item N3 (*Feelings of belonging*) will increase the number of items in the social domain from three items to four items. As three items are generally considered the bare minimum for a sub-scale, the psychometric robustness of the social domain has improved with this new additional item. However, it is important to note that anyone using the national items as part of a domain score will need to state this explicitly, so that no comparisons will be made with domain scores from studies reporting domain scores that do not contain the national items.

Four of five New Zealand national items belong to the psychological domain, indicating the importance of psychological aspects of QOL to New Zealanders. Similar to the Taiwanese and Hong Kong versions [10], one such item asks about the respect received from other people. Another item (*Feelings of belonging*) that was assigned to the social relationships domain could be a reflection of the influence of collectivism of Māori culture [23] on the dominant individualistic British culture [24]. Similarly, the item *Meeting expectation* may also be a reflection of Māori and Polynesian culture, where values and obligations are centred around the extended family and community [25].

While more than half of themes from the focus groups of Study 1 related to physical and environmental aspects, none of the new items generated from these themes remained after the various psychometric criteria had been applied. No new national items were therefore developed for the physical and environment domains. One theme that had emerged very strongly in the focus groups was access to New Zealand’s natural environment. Most New Zealanders are proud of the country’s landscape, with a clean and green image being part of the collective psyche [26]. However, none of the items about the natural environment and outdoor activities correlated with QOL or health and could therefore not be justified to be included. This does not necessarily imply that outdoor activities or activities in natural surroundings are not beneficial to quality of life and health as no information was gathered on actual frequency of such activities. Additionally, the importance of these facets was assessed in the context of many others, and there is the possibility that participants may have tended to take access to outdoors and nature for granted.

A general limitation of the two quantitative surveys was certainly the low response rate and representativeness of the sample. The response rates of 29% for Study 1 and 24% for Study 2 are not ideal but certainly not uncommon for health research of that kind in New Zealand [27]. Another issue was the demographic distribution in the two surveys. In both surveys, younger adults were underrepresented, which led to the decision to purposively target younger people for additional data. On a final note, these results should only be taken as preliminary and are only published here as work in progress. While we were able to make comparisons between participants who considered themselves as well and those who considered themselves as unwell, further testing of the national items will be required with populations that are clearly identified as unhealthy. This will provide a more stringent test of discriminant validity.

The New Zealand WHOQOL Group [28] monitors the use of the WHOQOL instruments in New Zealand. Please contact the first author for formal registration, or refer to cpcr.aut.ac.nz/new-zealand-whoqol or whoqol.org.nz. Users are encouraged to share their anonymised datasets to be added to an ongoing database of reference values, which all registered users are able to access and use for comparative purposes. The New Zealand WHOQOL Group also provides a user manual with reference values that are updated regularly. The authors may also be contacted for access to the dataset reported on in the present article. These files are also available on the journal’s website as supporting information (S1 and S2 Files).

## Supporting Information

S1 File Data for Study 2. This SPSS file contains ratings from a sample of 585 New Zealanders of importance data for the candidate national items shown in Table 2 as well as for the global QOL and health items.
(SAV)

S2 File Data for Study 3. This SPSS file contains ratings from a sample of 808 New Zealanders of WHOQOL-BREF items as well as the five national items listed in Table 3. Item columns are arranged by QOL domains. Also shown are the *g* facet scores and total QOL as well as the age categories used in Rasch analysis to explore DIF.
(SAV)
